# Osteochondritis Dissecans in the Medial Femoral Condyle: A Case Report and Review of the Role of Autogenous Mosaicplasty in Articular Cartilage Repair

**DOI:** 10.7759/cureus.75167

**Published:** 2024-12-05

**Authors:** Bader Aljadaan, Fahad Alotaibi, Sarah A Maghrabi, Sultan Mohammed F Alanazi, Faisal A Alqifari

**Affiliations:** 1 Orthopaedic Surgery, Prince Sultan Military Medical City, Riyadh, SAU; 2 Orthopaedic Surgery, King Saud University Medical City, Riyadh, SAU; 3 Orthopaedics, King Saud University, Riyadh, SAU

**Keywords:** arthroscopic surgery, autogenous mosaicplasty, joint function, medial femoral condyle, osteochondritis dissecans

## Abstract

Osteochondritis dissecans is a rare condition characterized by the deterioration of a small area of bone and cartilage without infection. Its exact cause is unclear, though factors such as abnormal bone development, joint pressure, repetitive injuries, inadequate blood supply, and genetic links have been observed. In this case, a 27-year-old woman experienced chronic right knee pain following a twisting injury, which led to reduced mobility and mild pain. Examination showed no swelling or tenderness, although X-rays and further imaging revealed irregularities in the medial femoral condyle, a 1.5 cm osteochondral defect, a loose fragment, and mild degenerative changes in the meniscus and partial ligament injuries. Post-surgical management included early gentle movements and a transition to running four months post-operatively. Follow-ups at 10 days and six weeks showed a full range of motion with no pain or complications. Successful graft integration and healing were confirmed without significant osteoarthritic changes, indicating effective joint preservation. This case illustrates the potential of autograft mosaicplasty in treating osteochondritis dissecans, underlining the importance of individualized treatment approaches to address articular cartilage injuries effectively. Long-term results and the factors contributing to the disorder remain areas for further research.

## Introduction

Osteochondritis dissecans (OCD) is a rare condition in which a small area of bone and its surrounding cartilage deteriorates without any infection. Essentially, a patch of bone and cartilage weakens and breaks down. Despite extensive research, the exact cause of this condition is still unclear. However, several factors are believed to contribute, including abnormal bone development, excessive pressure on the joint, repetitive minor injuries, and inadequate blood supply to the affected area [1]. There is also evidence to suggest that genetics may play a role, as multiple cases of knee osteochondritis dissecans have been observed within the same family, indicating a possible hereditary link [2]. This condition can affect various joints, but it is most commonly found in the knee, particularly in the medial and lateral femoral condyles [1]. Over 70% of cases occur on the posterior aspect of the medial femoral condyle, which seems to be especially vulnerable. In contrast, only 15% to 20% of cases affect the lower part of the lateral femoral condyle, and bilateral involvement in adults is extremely rare [2]. Treatment decisions for OCD are based on several key factors, including the patient’s skeletal maturity, the size and stability of the lesion, and the condition of the bone and cartilage connection. Younger patients, especially those with smaller lesions located in the medial femoral condyle, tend to have more favorable outcomes with conservative treatment, as their bones are still growing [1]. Success rates for non-surgical approaches range from 50% to as high as 94%, allowing the body to heal naturally without surgery [3]. However, when patients near skeletal maturity, or if the lesion is unstable, detached, or fails to respond to conservative treatments, surgical intervention becomes necessary [4,5]. Surgical options for OCD vary and include debridement, grafting, and techniques to introduce fresh cartilage cells into the damaged area [6]. The goal is always to preserve as much bone and cartilage as possible, but for larger or irreparable defects, surgeons may use autografts or allografts, secured with screws or other methods for stabilization [3]. Autogenous mosaicplasty, a technique that uses multiple small grafts rather than a single large one, has proven particularly effective for treating osteochondritis dissecans [7]. However, the use of autografts is limited by the amount of available donor material, which may be insufficient for larger lesions. The purpose of this study is to assess the effectiveness of different treatment approaches for osteochondritis dissecans, focusing on a comparison between conservative methods and surgical options such as mosaicplasty and grafting. The goal is to identify the best strategies for preserving joint function and preventing long-term damage while also investigating the influence of genetics and mechanical factors on the condition's development. A literature search was conducted to collect studies on osteochondritis dissecans in the medial femoral condyle and the role of autogenous mosaicplasty in articular cartilage repair. We searched PubMed and Scopus databases for articles in the field with no restriction to date, study type, or language and included 15 studies.

## Case presentation

A 27-year-old female presented at Prince Sultan Military Medical City with chronic right knee pain following a twisting injury. Two years prior, she began experiencing intermittent, activity-related knee pain that worsened with flexion. The pain was mild and improved with medication, without any grinding, instability, or sensation of the knee giving way. Her medical, surgical, and family history was unremarkable.

On examination, the right knee showed no tenderness or swelling, particularly around the medial and lateral joint lines. The range of motion was reduced (5 to 130 degrees) with mild pain at the end of flexion. Stability tests for the anterior cruciate ligament and varus and valgus stress were negative. A neurovascular examination of the lower extremities and assessments of the feet, hips, upper extremities, and spine showed no abnormalities.

**Figure 1:**
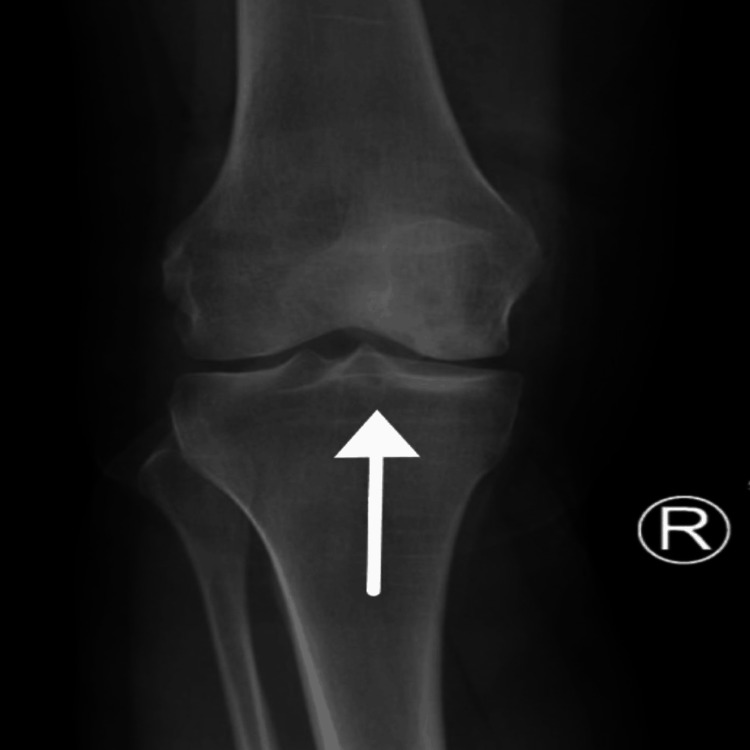
Pre-operatve X-ray of the right knee A white arrow highlights a radiolucent area on the anteroposterior aspect of the medial femoral condyle

X-ray imaging of the right knee displayed irregularities in the articular surface on the lateral side of the medial femoral condyle and joint fluid accumulation. A white arrow (Figure 1) highlights a radiolucent area on the medial femoral condyle, indicating a subchondral defect typical in OCD. The lesion appeared crescent-shaped, suggesting stage III or IV due to possible fragment detachment. Sclerosis around the fragment indicates chronicity, with an absence of calcification or ossification, suggesting that the loose body has not fully ossified. The lesion size ranged from a few millimeters to over a centimeter, which might require complex surgical intervention. The joint space was preserved, showing no secondary degenerative changes. Advanced imaging, such as MRI, was recommended for further assessment of cartilage damage.

Computed tomography confirmed a 1.5 cm osteochondral defect in the medial femoral condyle, with a displaced fragment of bone and cartilage in front of the posterior fat pad. MRI showed a large defect in the medial femoral condyle involving both bone and cartilage, with fluid buildup and degenerative changes in the medial meniscus. Signs of mild to moderate partial injury to the anterior cruciate ligament and a strain in the medial collateral ligament were also noted, confirming the diagnosis of OCD. 

**Figure 2:**
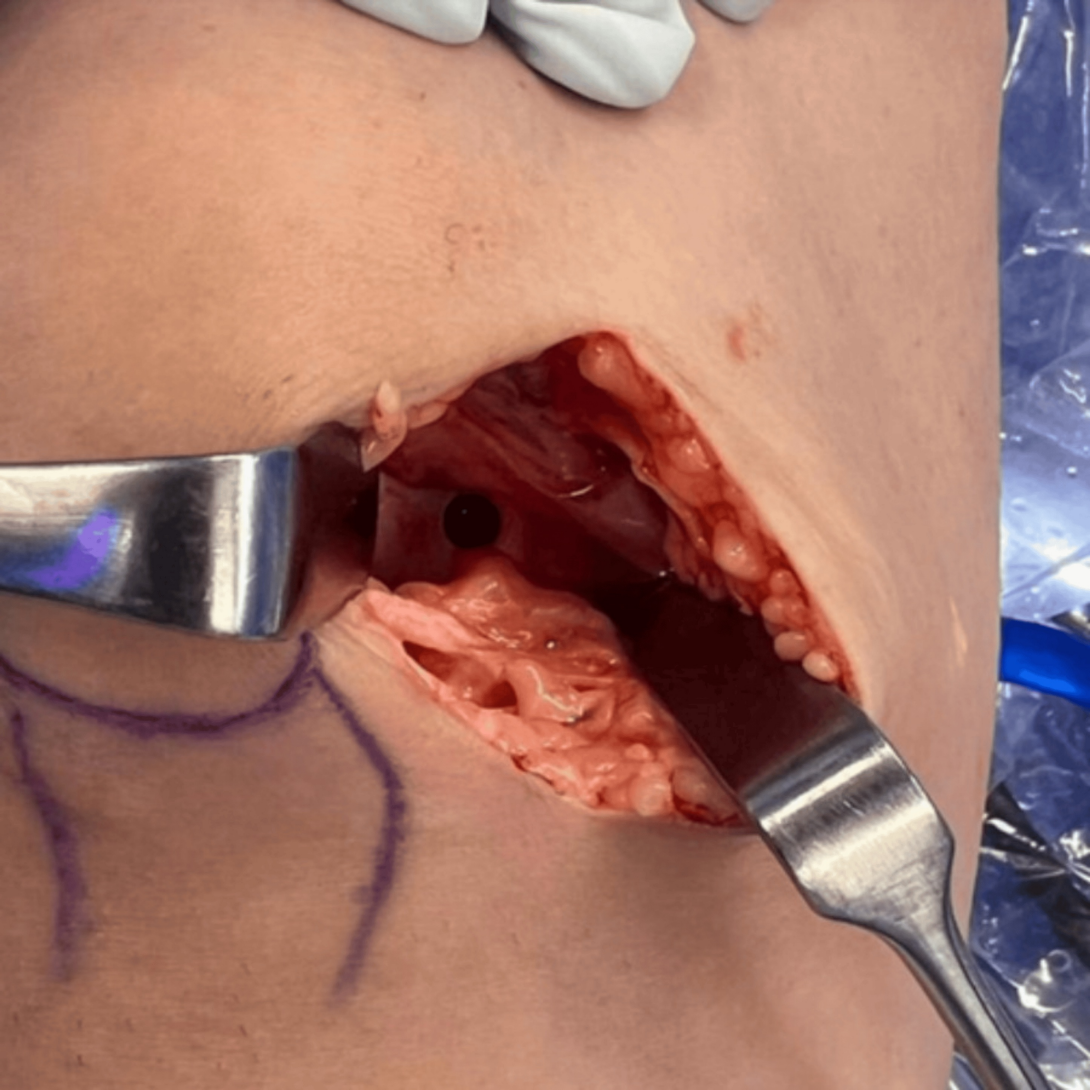
Intra-operative imaging A medial parapatellar approach was used, typical for mosaicplasty, with three cylindrical osteochondral grafts harvested from a non-weight-bearing area and inserted into the lesion

Surgery was planned using the mosaicplasty technique due to the severity of the defect. This procedure involved harvesting healthy bone and cartilage grafts from non-weight-bearing areas of the knee. The surgical field displayed an open arthrotomy, with the skin, subcutaneous tissue, and joint capsule retracted for better visualization. The OCD lesion on the medial femoral condyle presented as an irregular subchondral area, prepared for mosaicplasty. A medial parapatellar approach was employed, which is typical for this technique. Three cylindrical osteochondral grafts were taken from a non-weight-bearing area and inserted into the lesion (Figure 2) to restore the articular surface and preserve the natural curvature of the femoral condyle. After confirming a full range of motion without limitations, the knee was closed with sutures.

**Figure 3:**
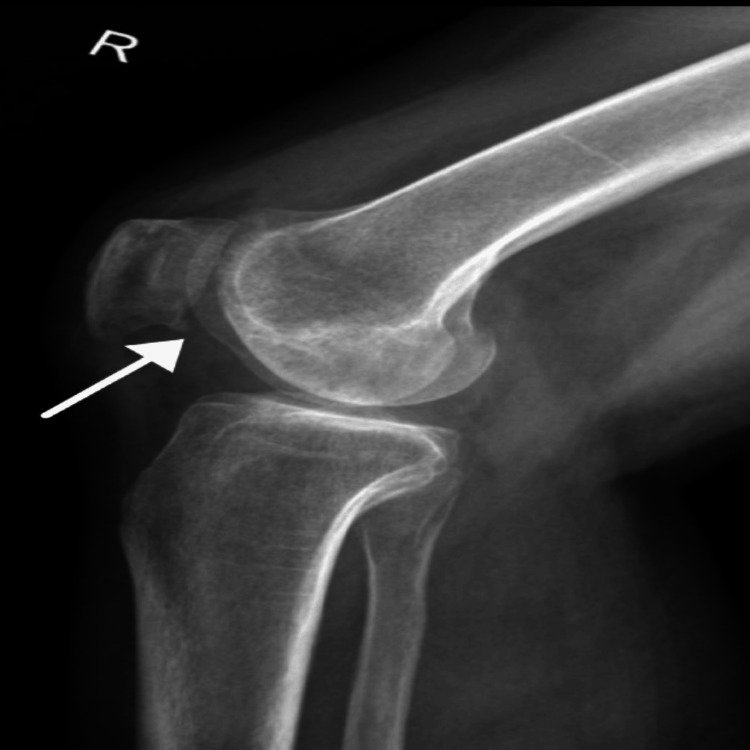
Post-Operative X-ray Findings Lateral view of the left knee after surgical repair The arrows indicate the graft site on the medial femoral condyle.

Post-surgery, the patient was instructed to begin gentle knee movement immediately, with joint-friendly activities like swimming and cycling for the first 2.5 months. Running was to be introduced at four months, with full activity expected by six months. Follow-up examinations at 10 days and six weeks post-surgery revealed full range of motion without pain or locking. The graft site on the medial femoral condyle (Figure 3) showed well-seated bone plugs from the mosaicplasty, with no displacement or osteoarthritic changes, and preserved joint space. Sclerotic changes around the graft site indicated bone healing and integration.

**Figure 4:**
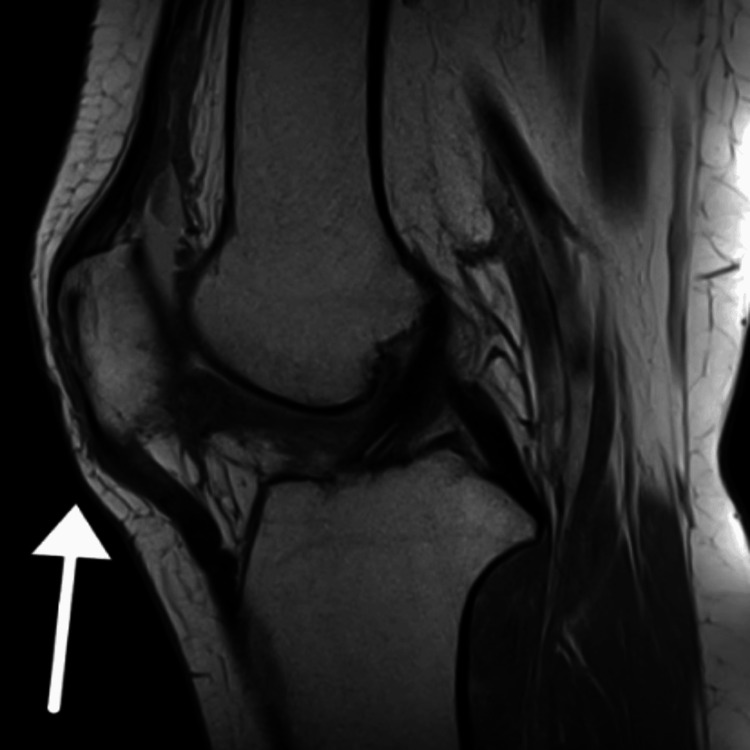
Post-Operative MRI Sagittal view of the knee The area highlighted by the arrow represents the location of the graft, showing improved articular contour. The appearance of normal bone marrow signal intensity around the graft suggests favorable healing, which aligns with the minimal joint effusion observed.

A sagittal MRI of the knee post-mosaicplasty revealed several findings, including the area of osteochondral graft placement, which showed improved articular contour and successful graft integration (Figure 4). The signal intensity near the repaired area was closer to that of normal bone marrow, indicating favorable healing. Additionally, minimal joint effusion was present, suggesting controlled post-operative inflammation.

## Discussion

The goal in treating OCD is to preserve the original bone and cartilage. In growing children, conservative treatments-such as limiting weight-bearing, immobilization, and activity modifications-can be effective, allowing natural healing without surgery [8,9]. Surgical options should be considered for younger patients with detached or unstable lesions, or those nearing the end of their growth period who do not respond to non-surgical methods. Surgery aims to stabilize the joint and prevent further damage as they transition into adulthood [3].

For lesions smaller than 2 cm, arthroscopic procedures like subchondral drilling, debridement, and fragment stabilization are typically recommended. In contrast, larger lesions or those with multiple loose bodies may require an open surgical approach, possibly using advanced techniques like autologous chondrocyte implantation or mosaicplasty [8].

Mosaicplasty, a technique introduced in 1985, involves harvesting cylindrical grafts of bone and cartilage from non-weight-bearing areas to rebuild damaged regions. It has shown high effectiveness, with success rates reaching up to 94% for surgical treatment of OCD lesions in the femoral condyle [8]. This method offers advantages such as being a one-step arthroscopic procedure and utilizing hyaline cartilage, which has superior mechanical properties compared to fibrocartilage. However, drawbacks include potential complications at donor sites, possible size mismatches, and limited areas available for graft harvesting [9].

In our case, the patient had an osteochondral defect on the lateral aspect of the medial femoral condyle, confirmed by arthroscopic examination. While trauma is commonly associated with OCD, factors like repetitive microtrauma and vascular disturbances contributing to bone necrosis are significant [10]. A systematic review from 2018 supported the mechanical hypothesis, linking injury or overuse to the condition. Genetic factors could not be confirmed in this case since the patient's family declined examination.

During follow-up, the patient showed significant improvement, achieving full range of motion, with no clicking, locking, or pain. Radiographic images indicated successful correction of the OCD defect and restoration of joint function. Positive outcomes were also reported in other studies, where patients treated with cylindrical osteochondral grafts experienced satisfactory results [4]. Additional research indicated satisfactory outcomes for OCD lesions ranging from 0.5 to 3.2 cm², suggesting that mosaicplasty is effective for a range of lesion sizes, particularly smaller defects [11].

A study involving 12 cases of OCD showed significant functional improvement with arthroscopic cylindrical autogenous osteochondral plugs, with no gaps between grafts and subchondral bone noted three months post-surgery [12]. Another study found better outcomes for medial condyle defects compared to those in the lateral condyle or patellofemoral region, highlighting the importance of defect location [11].

In a randomized study comparing 29 cases of mosaicplasty with 29 of microfracture for defects averaging 2.7 cm², mosaicplasty was found to yield significantly better outcomes after three years. Notably, biopsies from the mosaicplasty group showed no fibrocartilage, with 93% of patients reporting good results, compared to only 49% in the microfracture group [13].

Further studies comparing mosaicplasty with first-generation autologous chondrocyte grafts showed no significant difference in outcomes for defects averaging 3.7 cm² [14]. This finding was echoed by another study, reinforcing the comparable outcomes of mosaicplasty and first-generation chondrocyte grafts in treating osteochondral defects [15]. While contrasting studies reported better performance of first-generation chondrocyte grafts, they noted that larger defects (up to 12.2 cm²) and small diameter plugs used in mosaicplasty might influence outcomes, underscoring the need to consider defect size and graft characteristics when choosing a treatment approach for osteochondral lesions [16]. 

The 15 PubMed and Scopus articles that we reviewed are presented in Table 1.

**Table 1:** Characteristics of the studies included in the review OCD: Osteochondritis dissecans. ACI: autologous chondrocyte implantation

First author (Study year of publication)	Study type	Anatomical Context	Specific Findings	Radiographic Features of OCD	Clinical Correlation
Keyhani (2020) [1]	Case series	Focused on knee joint lesions with osteochondral defects.	Autogenous osteochondral grafting effectively restored joint stability and function in treated cases.	Imaging confirmed successful graft integration and joint contour restoration, with menial	Supports the use of autogenous osteochondral grafting as a viable treatment for knee OCD particularly.
Jeong (2013) [2]	A report of two sibling cases	Studied bilateral OCD of the femoral condyles in siblings, emphasizing genetic factors.	Highlighted the importance of early diagnosis and the role of conservative vs. surgical management.	Radiographs and MRI confirmed the bilateral OCD, with stable lesions in early stages and unstable lesions in advanced cases.	Suggests genetic predisposition in OCD and recommends regular monitoring.
Cahill (1995) [3]	Review article	Reviewed both adult forms of knee OCD.	Outlined varied outcomes based on lesion stability, recommending early intervention for better results.	Imaging studies showed better responses to conservative treatment in early-stage OCD.	Emphasizing early diagnosis and treatment to prevent lesion progression.
Kobayashi (2004) [4]	Case report	Focused on massive OCD lesions of the knee, specifically targeting surgical fixation techniques.	Found cylindrical osteochondral plugs stabilizing massive lesions, restoring joint function.	Radiographs showed successful plug integration, while surgical intervention while MRI confirmed continuity of cartilage and subchondral bone healing.	Supports using cylindrical plugs as a viable surgical option for massive OCI.
Wall & Von Stein (2003) [5]	Review article	Focused on juvenile OCD of the knee, discussing management challenges.	Discussed benefits of conservative vs. surgical management, recommending early surgery for severe cases.	Imaging findings Supported the need for early surgical intervention in advanced cases to prevent further joint damage.	Emphasizing the importance of early surgical intervention for better long-term joint health.
Gikas (2007) [6]	Comparative study	investigated biopsy timing after ACl in the knee joint and its histological outcomes.	Found that earlier biopsies yielded better histological outcomes suggesting, benefits for timely intervention.	Imaging findings correlated with histological results, showing better cartilage regeneration with earlier interventions^.^	Supports the need timely intervention ACI procedures to improve and gecartila.
Gudas (2009) [7]	Randomized controlled trial	Focused on knee OCD in children, comparing osteochondral autologous transplantation vs microfracture.	Both treatments were effective, but osteochondral transplantation provided quicker functional recovery.	Radiographs showed better defect filling and graft stability in osteochondral transplantation cases, while microfracture required longer healing times.	Suggests using osteochondral transplantation.
Roberti (2021) [9]	Review article	Focused on fresh osteochondral allografts for treating OCD in the knee.	Demonstrated promising outcomes, with grafts integrating well into the joint surface.	Imaging confirmed successful alignment and restoration of joint contours, showing the integration of grafts.	Supports using osteochondral allografts for restoring knee function, providing an effective treatment option for advanced OCD.
Gudas R. (2006) [10]	Randomized controlled trial	Focused on the treatment of osteochondral defects in athletes’ knees, comparing autologous transplantation and microfracture.	Found that autologous transplantation showed superior outcomes in terms of stability and defect.	Radiographs confirmed better graft integration and defect coverage in autologous transplantation compared to microfracture.	Supports using autologous transplantation.
Hangody L. (2008) [11]	Review article	Studied the technique and long-term outcomes of autologous osteochondral grafting in the knee joint.	Reported good long-term outcomes with high patient satisfaction.	Radiographs showed stable with no significant degradation over time.	Supports autogenous osteochondral grafting as a viable long-term solution for restoring knee function and structure in OCD patients.
Miura H. et al. (2007) [12]	Case series	Focused on cylindrical autogenous plugs for OCD treatment.	Found cylindrical plugs effective in restoring joint stability reducing symptoms.	MR confirmed plug integration, showing proper alignment and cartilage coverage.	Supports cylindrical autogenous plug as an effective surgical option for OCD management, especially in cases requiring arthroscopic intervention.
Gudas R. (2005) [13]	Randomized controlled trials	Focused on mosaic osteochondral transplantation vs. microfract for knee defects in young athletes.	Mosaicplasty showed better outcomes compared to microfracture, particularly in terms of functional recovery.	Imaging confirmed better defect filling cartilage coverage with mosaicplasty.	Supports using mosaicplasty for faster recovery and better defect coverage in young athletes with knee OCD.
Horas (2003) [14]	Clinical trial	Focused on cartilage repair in the knee joint, comparing ACl with osteochondral cylinder transplantation.	Both ACl osteochondral were effective but each had varying recovery profiles.	MR and radiographic of grafts, but recovery times differed between the techniques.	Suggests individualized treatment based on patient recovery time.
Dozin (2005) [15]	Clinical trial	Evaluated the effectiveness of ACl vs. mosaicplasty in knee cartilage repair across multiple centers.	Found mosaicplasty favorable for smaller defects, while ACl was better suited for larger lesions.	Imaging confirmed effective graft integration for both methods, with variations depending on lesion size and treatment approach.	Suggests mosaicplasty as the preferred treatment smaller defects.
Bentley (2003) [16]	Clinical trial	Focused on the comparative outcomes of ACl and mosaicplasty for treating knee osteochondral defects.	Found that ACl was better for larger defects, while mosaicplasty was more effective for smaller lesions.	Radiographic imaging confirmed better defect coverage with mosaicplasty in smaller lesions while ACl showed superior integration in larger defects.	Highlights the importance of lesion size.

## Conclusions

In osteochondral grafting, particularly autograft mosaicplasty, was effective in treating the patient's chronic knee pain related to an osteochondritis dissecans defect. The procedure successfully restored joint function and alleviated symptoms, demonstrating its utility in such cases. Continued follow-up and long-term monitoring will be crucial to assess the durability and longevity of the surgical outcome. This experience emphasizes the importance of individualized treatment approaches tailored to the patient's specific pathology and clinical presentation for achieving favorable results in articular cartilage injuries.
